# Breaking the mould: stakeholder insights into the shift from 2 + 4 to a 6-Year continuous medical curriculum in South Korea

**DOI:** 10.1186/s12909-025-07998-8

**Published:** 2025-10-10

**Authors:** Yoonjung Lee, Jwa-Seop Shin, Wan Beom Park, Hyun Bae Yoon

**Affiliations:** 1 Department of Human Systems Medicine, Seoul National University College of Medicine, 103, Daehak-ro, Jongno-gu, Seoul, South Korea 03080 Republic of Korea; 2 Department of Medical Education, Seoul National University College of Medicine, Seoul, South Korea Republic of Korea; 3 Department of Internal Medicine, Seoul National University College of Medicine, Seoul, South Korea Republic of Korea

**Keywords:** Curriculum reform, Competency-based medical education, 6-year continuous medical curriculum, Stakeholder engagement, South korea

## Abstract

**Background:**

In response to global reforms emphasizing competency-based learning and integrated curricula, South Korea is transitioning from its traditional 2 + 4 medical education model to a 6-year continuous medical curriculum. This shift addresses challenges such as fragmented learning pathways and insufficient integration between foundational and clinical training, which have limited the development of professional competencies and holistic student engagement. However, empirical evidence on stakeholder perspectives regarding this transition remains limited. This study aimed to examine faculty and student views on the proposed reform to inform its implementation and contribute to broader discussions on medical education transformation.

**Methods:**

A mixed-method study was conducted at Seoul National University College of Medicine. Quantitative data were collected from an online survey completed by 142 faculty members and 133 medical students. Qualitative data were gathered from two focus group interviews with fifteen students from both pre-medical and clinical phases. Survey responses were analyzed using Welch’s t-tests, and thematic analysis was applied to focus group interview data.

**Results:**

Quantitative analysis revealed significant differences between faculty and student perceptions of the proposed 6-year continuous medical curriculum. Faculty reported higher scores for perceived needs (M = 4.05 vs. 2.79, *p* < .001), continuity, motivation, and curriculum supplementation (all *p* < .01). Students expressed greater concerns about curriculum overcrowding, academic burden, reduced interdisciplinary learning, and stress (all *p* < .01), and prioritized vacation time (M = 4.39 vs. 3.82, *p* < .01), extracurricular support (M = 4.14 vs. 3.52, *p* < .01), and pass/fail grading (M = 4.09 vs. 3.36, *p* < .01). Faculty emphasized integration of spiral and repetitive curriculum (M = 3.55 vs. 3.49, *p* = .02) and new content such as data science and health systems science (all *p* < .01). Thematic analysis identified student expectations for early integration of clinical and foundational subjects, balanced workload, and structured support systems, alongside concerns about marginalization of the pre-medical phase and prolonged stress.

**Conclusion:**

The transition to a 6-year continuous medical curriculum requires a comprehensive approach that engages key stakeholders, particularly students. Balancing educational innovation with curriculum manageability and student well-being is essential. Participatory design, phased implementation, and robust support systems are needed to ensure sustainable and adaptive curriculum reform.

**Supplementary Information:**

The online version contains supplementary material available at 10.1186/s12909-025-07998-8.

## Introduction

Medical education worldwide is undergoing profound transformation in response to rapid advances in medical knowledge and evolving healthcare environments [1]. To meet the demands of modern medical practice, curricula across diverse educational contexts are being restructured to emphasize key competencies such as clinical reasoning [2], communication skills [3], and social accountability [4]. These reforms reflect the principles of competency-based medical education (CBME), which promotes developmental progression [5], continuous learning and assessment [6], and early clinical exposure [7, 8] as essential elements for preparing future physicians.

Within this context, South Korea has traditionally adhered to a “2 + 4” medical education model, comprising two years of pre-medical coursework followed by four years of medical education. While this structure was originally designed to bridge gaps in pre-university preparation [9, 10], it has faced growing criticism for its rigid separation between foundational and clinical learning [11], delayed clinical exposure, and limited integration of knowledge and skills [12]. Also, academic performance during the pre-medical phase has minimal influence on residency applications, which reduces motivation and engagement during the early stages of training [13]. Compressing clinical rotations into the latter four years has further compounded challenges in integrating foundational knowledge and clinical practice, thereby impeding the development of professional identity and holistic learning. In response to these structural and pedagogical limitations, South Korea is preparing to transition to a continuous six-year integrated medical curriculum. In accordance with global CBME principles, the six-year medical curriculum integrates liberal arts, basic sciences, and clinical experiences in a progressive spiral model, which revisits core concepts with increasing complexity to promote interdisciplinary learning, early clinical exposure, and comprehensive competency development for lifelong learning [14].

Empirical evidences from integrated curricula in other contexts had demonstrated improvements in student engagement, motivation, and professional identity formation, as well as enhanced preparedness for clinical practice [15, 16]. However, the successful implementation of such reforms necessitates careful adaptation to the specific educational and healthcare contexts in which they are applied [17]. While the importance of engaging both faculty and students in curriculum reform processes is widely acknowledged, studies that have actively incorporated both perspectives into curriculum redesign are scarce, and research explicitly examining differences in perceptions between faculty and students during the reform process is also limited [18, 19]. Gaining insights into these potential differences is essential for designing and implementing reforms that effectively reflect the needs and expectations of all stakeholders. Therefore, our study aimed to examine the needs, expectations, and concerns of both students and faculty members as the principal stakeholders of the 6-year continuous medical curriculum to prepare and develop a more effective medical education system. Furthermore, the findings are expected not only to advance medical education in Korea but also provide valuable insights that could influence global medical education policy and practice. Moreover, as similar shifts from segmented to continuous curricula are being explored in various countries, the findings of this study may serve as a reference point for guiding comparable reforms elsewhere, thereby reinforcing its significance within the broader discourse on global medical education.

## Methods

This study was conducted at a single institution, Seoul National University College of Medicine, which is transitioning from a 2 + 4 to a 6-year continuous medical curriculum.

### Participants

A total of 534 faculty members and 272 medical students were invited to participate in the survey. Of these, 142 faculty members (26.6%)and 133 students (48.9%)completed the final version of the survey (Table 1). Only fully completed surveys were included in the final analysis to ensure data integrity, as partial responses might omit critical information required for valid comparison. For the qualitative component, fifteen students participated in focus group interviews, including pre-medical and clinical-phase students (Table 2).

**Table 1:** Demographic information of the online survey participants

Variable	No.(%)
Total	**275**
Faculty members	142 (51.6)
Specialty	
Basic medical science	39 (27.5)
Clinical medicine	96 (67.6)
Humanities and social medicine	7 (5)
Pre-medical student teaching experience	
Yes	44 (31)
No	98 (69)
Student	133 (48.4)
Gender	
Male	75 (56.4)
Female	58 (43.6)
Year in the program	
Third	63 (47.4)
Fourth	70 (52.6)

**Table 2:** Demographic information of the participants in the focus group interviews (FGIs)

Variable	No.
Total	15
Gender	
Male	9
Female	6
Year in the program	
First	3
Second	4
Fourth	3
Fifth	3
Sixth	2

### Data collection

The Office of Medical Education conducted all survey data collection. The survey questions were initially piloted with a curriculum development committee comprising twenty-four academic leaders and department chairs, of whom twenty completed the pilot survey. Feedback was used to assess internal consistency and revise item wording for clarity and contextual relevance. The final survey included six domains: new educational environment to be prepared, new educational content to be prepared, principles to be followed when converting to the 6-year continuous medical curriculum, needs of the 6-year continuous medical curriculum, expectations toward the 6-year continuous medical curriculum, and worries about the 6-year continuous medical curriculum. Items were rated on a 5-point Likert scale (1 = strongly disagree to 5 = strongly agree), and faculty respondents also completed open-ended questions regarding the average distribution of phases during the total 6-year continuous medical curriculum and the average distribution of themes by year. In the qualitative analysis, two focus group interviews (FGIs) were conducted with students from pre-medical (first and second years) and clinical phase (fourth to sixth years). A total of sixteen students were scheduled for participation. Two were purposively recruited from the student representative council, and the remaining fourteen were recruited via snowball sampling to enhance participant diversity. While this approach facilitated the inclusion of a wide range of perspectives, it may also have introduced potential self-selection bias, as students with stronger interest or opinions regarding the curriculum reform might have been more likely to participate. One student withdrew on the day of the session, resulting in a final total of fifteen participants. Each FGI lasted 60–90 min, was audio-recorded, transcribed verbatim, and anonymized. The Office of Medical Education provided anonymized online survey data (Excel file) and FGI data (Word file) to the researchers for analysis.

### Data analysis

Quantitative data were analyzed using IBM SPSS Statistics version 26. Descriptive statistics were computed for each survey item. The Shapiro-Wilk test and Levene’s test were used to assess normality and homogeneity of variances, respectively. Welch’s t-tests were applied where variances were unequal, and significant differences between faculty and student responses were identified using these tests. A two-tailed p-value < 0.05 was considered statistically significant.

Qualitative data from focus group interview (FGI) transcripts were analyzed using thematic analysis in ATLAS.ti version 24. Two researchers independently performed open coding and developed sub-themes through discussion. To ensure robustness and reliability, the final analysis was refined through triangulation [20], with discrepancies between the two coders resolved by a third researcher to ensure the validity of the coding and themes.

## RESULTS

### Quantitative analysis

#### Survey results regarding the 6-year continuous medical curriculum

##### Needs, expectations, and worries

Table 3 summarized group differences in needs, expectations, and worries regarding the 6-year continuous medical curriculum between faculty members and students. Faculty members reported significantly higher mean scores than students for perceived needs (M = 4.05, SD = 0.95 vs. M = 2.79, SD = 1.16), t(246.69) = 9.84, *p* <.001, and consistently higher scores for continuity of pre-medical and medical education, motivation and responsibility, sense of belonging, and curriculum supplementation (all *p* <.01). In contrast, students reported higher mean scores than faculty members for worries, including curriculum overcrowding, increased academic burden, reduced interdisciplinary opportunities, and intensified competition (all *p* <.01). Faculty members reported higher mean scores than students for concerns about insufficient classrooms and faculty members, t(260.76) = 2.22, *p* =.02.

**Table 3:** Comparative analysis of faculty members and student perspectives on the 6-year continuous medical curriculum: needs, expectations, and concerns

Factor	Contents	Position	Mean ± SD	t(df)	*p*-value
Needs of the 6-year continuous medical curriculum.	Needs	Faculty	4.05 ± 0.95	9.84(246.69)	< 0.001
		Student	2.79 ± 1.16		
Expectations toward the 6-year continuous medical curriculum	Continuity of pre-medical and medical education	Faculty	4.28 ± 0.85	7.83(238.03)	< 0.01
		Student	3.31 ± 1.21		
	Promoting students’ learning motivation and responsibility during the first and second years	Faculty	4.29 ± 0.74	8.80(201.28)	< 0.01
		Student	3.13 ± 1.38		
	Improvement of sense of belonging	Faculty	4.28 ± 0.84	10.75(216.38)	< 0.01
		Student	2.79 ± 1.40		
	Supplementation of required education and un-overcrowded curriculum	Faculty	3.99 ± 1.06	3.44(250.01)	< 0.01
		Student	3.49 ± 1.37		
Worries about the 6-year continuous medical curriculum	Overcrowded 6-year curriculum	Faculty	3.33 ± 1.16	−7.39(271.32)	< 0.01
		Student	4.28 ± 1.04		
	Increased education beyond the student level	Faculty	2.97 ± 1.22	−5.04(272.92)	< 0.01
		Student	3.68 ± 1.19		
	Reduction of opportunity to explore other fields and interdisciplinary opportunities	Faculty	3.49 ± 1.16	−3.67(271.11)	**<** 0.01
		Student	4.01 ± 1.20		
	Intensifying student competition	Faculty	3.42 ± 1.16	−5.21(270.43)	< 0.01
		Student	4.14 ± 1.21		
	Insufficient classrooms and faculty members	Faculty	3.45 ± 1.14	2.22(260.76)	0.02
		Student	3.09 ± 1.33		

*Responses were rated on a 5-point Likert scale (1 = strongly disagree to 5 = strongly agree). Welch’s t-test was applied due to violation of the homogeneity of variances assumption.

##### Principles for conversion

 Table 4 showed group differences in perceptions of principles to be followed when converting to the 6-year continuous medical curriculum. Faculty members reported higher mean scores than students for spiral and repetitive curriculum (M = 3.55, SD = 1.08 vs. M = 3.49, SD = 1.02), t(212.4) = 2.21, *p* =.02, and reflecting university characteristics (M = 3.61, SD = 1.00 vs. M = 3.51, SD = 1.09), t(182.7) = 4.44, *p* <.01. Students reported higher mean scores than faculty members for guaranteed vacation (M = 4.39, SD = 0.93 vs. M = 3.82, SD = 0.96) and support for extracurricular activities (M = 4.14, SD = 0.92 vs. M = 3.52, SD = 1.00), both t-values = −4.20, *p* <.01.

**Table 4:** Comparative of faculty and student perspectives on principles for 6-year continuous medical curriculum

Factor	Contents	Position	Mean ± SD	t(df)	*p*-value
Principles to be followed when converting to the 6-year continuous medical curriculum	Spiral and repetitive curriculum	Faculty	3.55 ± 1.08	2.21(212.4)	0.02
		Student	3.49 ± 1.02		
	Guaranteed vacation	Faculty	3.82 ± 0.96	−4.20(208.9)	< 0.01
		Student	4.39 ± 0.93		
	Support for extracurricular activities such as club activities	Faculty	3.52 ± 1.00	−4.20(211.1)	< 0.01
		Student	4.14 ± 0.92		
	Reflecting the characteristics of the university	Faculty	3.61 ± 1.00	4.44(182.7)	< 0.01
		Student	3.51 ± 1.09		

*Responses were rated on a 5-point Likert scale (1 = strongly disagree to 5 = strongly agree). Welch’s t-test was applied due to violation of the homogeneity of variances assumption.

##### New educational environment and content

 Comparative analysis of faculty and student perspectives on the 6-year continuous medical curriculum: the new educational environment and content to be prepared showed statistically significant differences between the two groups (Table 5). Faculty members reported higher mean scores than students for new student selection system (M = 3.73, SD = 0.97 vs. M = 3.39, SD = 1.01), t(269.45) = 4.78, *p* <.01, and customized 6-year supervisor system (M = 3.62, SD = 0.74 vs. M = 3.35, SD = 0.83), t(258.13) = 2.41, *p* =.01. Conversely, students reported higher mean scores than faculty members for the expansion of the pass/fail evaluation system (M = 4.09, SD = 0.99 vs. M = 3.36, SD = 1.14), t(272.29) = −3.33, *p* <.01. Faculty members also reported higher mean scores for the importance of preparing new educational content, including data science, metaverse, health systems science (HSS), creativity and interdisciplinary opportunities, basic clinical competency, equality of human rights, communication and leadership, global competency, and basic medical topics such as embryology (all *p* <.01.

**Table 5:** Comparative analysis of faculty and student perspectives on the 6-year curriculum: new educational environment and content to be prepared

Factor	Contents	Position	Mean ± SD	t(df)	*p*-value
New educational environment to be prepared	New student selection system	Faculty	3.73 ± 0.97	4.78(269.45)	< 0.01
		Student	3.39 ± 1.01		
	Customized 6-year supervisor system	Faculty	3.62 ± 0.74	2.41(258.13)	0.01
		Student	3.35 ± 0.83		
	Expansion of the pass/fail evaluation system	Faculty	3.36 ± 1.14	−3.33(272.29)	< 0.01
		Student	4.09 ± 0.99		
New educational content to be prepared	Data science	Faculty	3.43 ± 0.75	3.42(248.84)	< 0.01
		Student	3.10 ± 0.79		
	Metaverse	Faculty	3.12 ± 0.97	3.57 (250.10)	< 0.01
		Student	2.85 ± 1.20		
	Health systems science (HSS)	Faculty	3.83 ± 0.73	3.32 (248.73)	< 0.01
		Student	3.35 ± 1.01		
	Creativity and interdisciplinary opportunities	Faculty	3.73 ± 0.95	4.57 (253.16)	< 0.01
		Student	3.39 ± 1.07		
	Basic clinical competency	Faculty	4.24 ± 0.63	5.40 (229.86)	< 0.01
		Student	3.56 ± 1.11		
	Equality of human rights, including the socially underprivileged	Faculty	3.77 ± 0.89	4.47 (214.67)	< 0.01
		Student	3.28 ± 1.32		
	Communication and leadership	Faculty	4.09 ± 0.61	5.41 (206.38)	< 0.01
		Student	3.54 ± 1.05		
	Global competency	Faculty	3.93 ± 0.85	3.31 (233.47)	< 0.01
		Student	3.61 ± 0.99		
	Basic medical topics such as embryology	Faculty	3.30 ± 0.85	4.42 (256.69)	< 0.01
		Student	2.8 ± 1.12		

*Responses were rated on a 5-point Likert scale (1 = strongly disagree to 5 = strongly agree). Welch’s t-test was applied due to violation of the homogeneity of variances assumption.

#### Open-ended questions regarding the average distribution of phases during the total 6-year continuous medical curriculum and the average distribution of themes by year

**Fig. 1:**
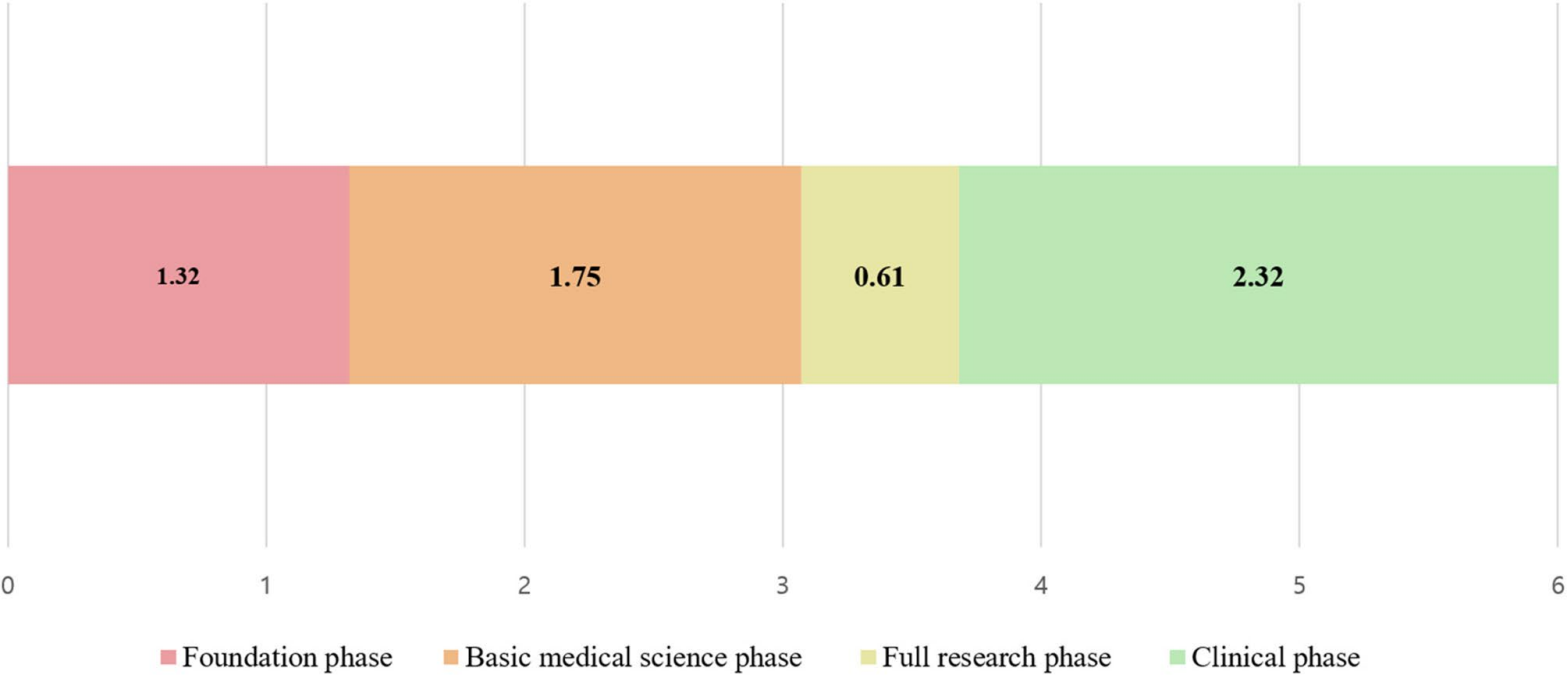
Average distribution of phases during the total 6-year curriculum

**Fig. 2:**
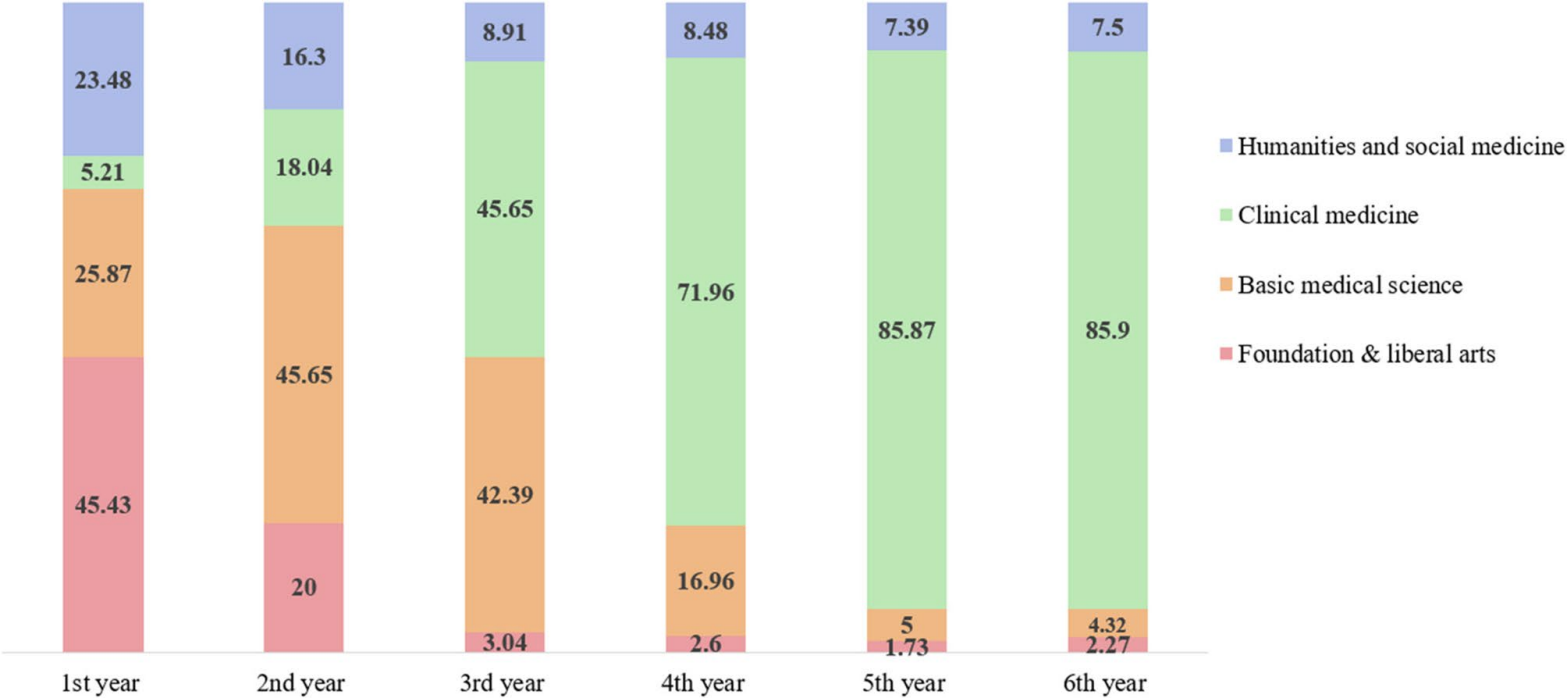
Average distribution of themes by year

Faculty members’ average responses to the question of how they would allocate the 6-year continuous medical curriculum for various phases were 1.32, 1.75, 0.61, and 2.32 years for the foundational phase, basic sciences phase, full research phase, and clinical phase, respectively (Fig. 1). Figure 2 shows the extent to which the themes would be addressed in each academic year for 6 years, excluding the research theme. For the foundational phase and medical humanities and social medicine, the proportion of responses appeared to decrease on average from the first to sixth year, whereas for clinical medicine, the proportion increased. For basic medical science, the proportion gradually increased from the first to the third year and gradually decreased from the fourth to sixth year.

### Qualitative analysis

Thematic analysis of focus group interviews (FGIs) with students revealed two overarching themes related to the 6-year continuous medical curriculum: (1) Expectations and (2) Concerns. Table 6 summarized the results of our study.

**Table 6:** Key themes, sub-themes, and representative quotes from qualitative analysis of student FGIs

Theme	Sub-theme	Quote	Student Year
Expectations	Early introduction of clinical and foundational subjects	“Starting clinical reasoning early will help us prepare as future doctors from the first year.”	Year 5 (clinical-phase)
		“A longer curriculum means more time to solidify the fundamentals before clinical training.”	Year 2 (pre-medical)
	Balancing academic rigor with personal development	“Vacations should allow rest and personal growth.”	Year 1 (pre-medical)
		“Limited vacation time constrained meaningful research and personal development”	Year 4 (clinical-phase)
	Structured academic support and guidance	“The new curriculum should provide stronger peer networks and academic support from the start.”	Year 2 (pre-medical)
		“It should include structured guidance for students struggling academically.”	Year 4 (clinical-phase)
Concerns	Loss of value in the pre-medical phase	“Pre-med let me explore clubs, meet diverse peers, and find interests beyond medicine.”	Year 1 (pre-medical)
		“It was essential for defining the kind of doctor and person I want to be.”	Year 6 (clinical-phase)
		“Liberal arts may be overshadowed by clinical courses.”	Year 5 (clinical-phase)
	Increased academic burden and stress	“Adding new subjects will overcrowd the curriculum and increase pressure.”	Year 4 (clinical-phase)
		“Continuous evaluation over six years will intensify stress and competition.”	Year 2 (pre-medical)
		“Switch to pass/no-pass grading to reduce stress.”	Year 6 (clinical-phase)

#### Theme 1: expectations for the 6-year continuous medical curriculum

##### Sub-theme 1.1: early introduction of clinical and foundational subjects

Students, particularly those in the clinical phase, anticipated that introducing advanced subjects such as clinical reasoning and basic sciences earlier would strengthen foundational knowledge and extend exposure to clinical thinking. One clinical-phase student shared, “Starting clinical reasoning early will help us prepare as future doctors from the first year” (Year 5, clinical-phase), while a pre-medical student noted, “A longer curriculum means more time to solidify the fundamentals before clinical training” (Year 2, pre-medical).

##### Sub-theme 1.2: balancing academic rigor with personal development

Students emphasized the need to balance demanding coursework with opportunities for rest, personal growth, and exploration of interests. A pre-medical student expressed, “Vacations should allow rest and personal growth” (Year 1, pre-medical), and a clinical-phase student noted, “Limited vacation time constrained meaningful research and personal development” (Year 4, clinical-phase).

##### Sub-theme 1.3: structured academic support and guidance

Pre-medical students highlighted the current lack of structure and connection during the early years, but expressed hope for stronger support and engagement under the new curriculum. One student commented, “The new curriculum should provide stronger peer networks and academic support from the start” (Year 2, pre-medical), while a clinical-phase student added, “It should include structured guidance for students struggling academically” **(**Year 4, clinical-phase).

#### Theme 2: concerns about the 6-year continuous medical curriculum

##### Sub-theme 2.1: loss of value in the pre-medical phase

Students, especially those in the pre-medical phase, valued this period as an opportunity for personal growth, extracurricular engagement, and identity formation. A pre-medical student shared, “Pre-med let me explore clubs, meet diverse peers, and find interests beyond medicine” (Year 1, pre-medical). A clinical-phase student remarked, “It was essential for defining the kind of doctor and person I want to be” (Year 6, clinical-phase). Another warned, “Liberal arts may be overshadowed by clinical courses” (Year 5, clinical-phase).

##### Sub-theme 2.2: increased academic burden and prolonged stress

Students feared curriculum overcrowding and stress from prolonged evaluation periods. A clinical-phase student cautioned, “Adding new subjects will overcrowd the curriculum and increase pressure” (Year 4, clinical-phase), while a pre-medical student said, “Continuous evaluation over six years will intensify stress and competition” (Year 2, pre-medical). Some suggested grading reforms, such as, “Switch to pass/no-pass grading to reduce stress” (Year 6, clinical-phase).

## Discussion

Our study showed the differing perspectives of faculty members and students regarding the transition from the traditional 2 + 4 model to 6-year continuous medical curriculum in South Korea. Like previous study, faculty largely viewed the reform as an opportunity to strengthen continuity [21], enhance student engagement [22], and improve educational quality [23]. In contrast, students voiced concerns about potential curriculum overcrowding, heightened academic stress, and diminished opportunities for interdisciplinary learning and personal development [24–27].

Several limitations should be considered in interpreting these findings. The study was conducted at a single institution, which may limit generalizability. The reliance on self-reported data from surveys and focus group interviews introduces the possibility of response bias, as participants may have provided socially desirable or selective responses. In addition, the absence of longitudinal tracking restricts the ability to examine how perceptions evolve over the course of curriculum implementation. Future longitudinal studies are needed to determine whether students’ initial concerns—such as curriculum overcrowding, heightened stress, and reduced interdisciplinary opportunities—persist, diminish, or change after they have experienced the full 6-year continuous medical curriculum. Despite these limitations, the integration of quantitative and qualitative methods provided complementary perspectives, offering a more comprehensive understanding of stakeholder views and a valuable foundation for future research.

Faculty members suggested a structured progression within the 6-year continuous medical curriculum, with early emphasis on foundational and basic sciences followed by increased clinical immersion in later years. While medical humanities and social medicine were emphasized at the outset, their prominence appeared to diminish over time, giving way to greater focus on clinical medicine. This pattern aligns with a spiral curriculum model, where key concepts are introduced early, revisited with increasing complexity, and ultimately integrated into clinical practice [28, 29]. However, such sequencing may not fully address student expectations for balanced workload, sustained personal development, and a holistic educational experience. Maintaining an emphasis on foundational and humanistic content throughout the program could help reconcile these differing perspectives [30].

The divergent priorities of faculty and students were also evident in specific concerns (Table 7). Faculty members emphasized continuity, resource readiness, and integration of emerging areas such as data science and global competencies, whereas students prioritized adequate vacation time, extracurricular opportunities, and flexible scheduling, alongside concerns about academic stress and reduced interdisciplinary engagement. Translating these findings into actionable strategies requires a phased implementation plan that allows for pilot testing and iterative adjustment; the establishment of formal student–faculty co-design mechanisms to ensure reforms reflect diverse perspectives; systematic monitoring of student workload and well-being; and targeted faculty development to strengthen capacity for delivering integrated, learner-centered curricula. Robust student support systems—including academic advising, mentoring, and mental health resources—are also critical for mitigating stress and promoting resilience [31–33].

**Table 7:** Summary of stakeholder perspectives and recommendations

Aspect	Faculty Views	Student Views	Curriculum Recommendations
Overall Perception	Support for reform to enhance continuity, engagement, and quality	Concerns over workload, stress, reduced interdisciplinary opportunities	Inclusive co-design, phased implementation with feedback
Academic Priorities	Emphasis on clinical integration, faculty preparation, resource allocation	Emphasis on pre-medical phase, vacation, extracurricular support	Balance clinical integration with foundational sciences and personal growth
Key Concerns	Infrastructure readiness, faculty development needs	Academic stress, mental health, competition	Strengthen advising, mentoring, mental health support
Preferred Features	Spiral curriculum, university-specific elements	Early clinical exposure, flexible scheduling, holistic support	Pilot testing, continuous refinement

International experiences, such as Singapore’s integrated curriculum and the UK’s spiral model, illustrate how longitudinal structures can foster deeper learning and professional identity formation when supported by sufficient resources, faculty preparation, and comprehensive student support [34–36]. Direct transfer of such models to the Korean context, however, must be approached with caution given the examination-driven culture and emphasis on academic achievement Aligning reforms with institutional capacity, creating dedicated opportunities for faculty training, and embedding ongoing evaluation processes will be essential to ensure operational feasibility and educational relevance. These steps are consistent with the principles of competency-based medical education (CBME), which prioritize learner-centeredness, flexible progression, and formative assessment [39–42]. Addressing systemic barriers such as entrenched practices and limited institutional flexibility will require context-specific solutions, collaborative curriculum design, and sustained evaluation.

Future research should extend these findings through multi-institutional, longitudinal, and mixed-methods approaches to track changes in stakeholder perspectives and assess the long-term impact of curricular reform on educational outcomes, professional identity formation, and student well-being. Such work should also evaluate effects on faculty workload, instructional quality, and resilience, using structured evaluation frameworks such as CIPP to capture both intended and unintended consequences.

## Conclusion

The transition to a 6-year continuous medical curriculum offers significant opportunities for advancing medical education, yet it also introduces substantial challenges that require deliberate planning and inclusive stakeholder engagement. This study emphasizes the necessity of finding a balance between educational innovation and the realities of implementation, while safeguarding student well-being and supporting faculty readiness. Actively involving students and faculty in co-design processes, supported by phased implementation and systematic feedback mechanisms, will be key to achieving a smooth transition and fostering a sustainable model. The experience in Korea not only underscores the potential of a participatory, evidence-informed approach but also reveals the complexities and institutional hurdles that may arise when reforms are pursued without adequate preparation. Moving forward, it will be crucial to focus on inclusivity, adaptability, and long-term sustainability to build an educational framework that effectively supports the evolving needs of both learners and educators.

## Supplementary Information

Supplementary Material 1

## Data Availability

The datasets used and/or analyzed during the current study are available from the corresponding author on reasonable request.
