# Supplementary material for: Breaking the mould: stakeholder insights into the shift from 2 + 4 to a 6-Year continuous medical curriculum in South Korea
Source: BMC Med Educ. 2025 Oct 10;25:1399. doi: 10.1186/s12909-025-07998-8 (PMC12512277; doi:10.1186/s12909-025-07998-8)
Supplement: Supplementary file 1 — Supplementary Material 1 [file 12909_2025_7998_MOESM1_ESM.docx]

**Survey questions**

1. What do you think about the necessity of transitioning to the continuous 6-year medical curriculum? Please rate from 1 to 5. (1=strongly disagree to 5=Strongly agree)

2. This question asks for your opinion on the expectation when transitioning to the continuous 6-year medical curriculum. Please select the appropriate option. (1=strongly disagree to 5=Strongly agree)

1) Continuity of pre-medical and medical education

2) Promoting students’ learning motivation and responsibility during the first and second years

3) Improvement of sense of belonging

4) Supplementation of required education and un-overcrowded curriculum

5) Guaranteed vacation

3. This question asks for your opinion on the worries when transitioning to the continuous 6-year medical curriculum. Please select the appropriate option. (1=strongly disagree to 5=Strongly agree)

1) Overcrowded 6-year curriculum

2) Increased education beyond the student level

3) Reduction of opportunity to explore other fields and interdisciplinary opportunities

4) Intensifying student competition

5) Insufficient classrooms and faculty members

4. This question asks about the principles that should be adhered to if the transition to the continuous 6-year medical curriculum is implemented. Please check your thoughts on the respective items. (1=strongly disagree to 5=Strongly agree)

1) Spiral and repetitive curriculum

2) Diverse curriculum according to student career interests

3) Guaranteed vacation

4) Support for extracurricular activities such as club activities

5) Reflecting the characteristics of the university

5. When transition to the six-year medical curriculum, please check your opinion on the new educational environment that need to be prepared in the list. (1=strongly disagree to 5=Strongly agree)

1) Link between main and multiple campuses

2) New student selection system

3) Customized 6-year supervisor system

4) Expansion of the pass/fail evaluation system

6. When transition to the six-year medical curriculum, please check your opinion on the new educational contents to be prepared in the list. (1=strongly disagree to 5=Strongly agree)

1) Data science

2) Metaverse

3) Health systems science (HSS)

4) Creativity and interdisciplinary opportunities

5) Basic clinical competency

6) Equality of human rights, including the socially underprivileged

7) Communication and leadership

8) Global competency

[Faculty only]7. In regard to the distribution of phases, transitioning to the 6-year continuous medical curriculum should fundamentally integrate foundation, basic medical science, full research, and clinical phase in a spiral, vertically integrated manner throughout the entire 6 years. Considering the structure of the 6-year curriculum into 1) Foundation phase, 2) Basic Medical Science phase, 3) Full Research phase, and 4) Clinical phase, please write the appropriate duration for each phase.

1) Foundation phase:

2) Basic Medical Science phase:

3) Full Research phase:

4) Clinical phase:

[Faculty only] 8. When transitioning to the continuous 6-year medical curriculum designed as a spiral curriculum, which aligns with global trends in medical education, please provide the average distribution of themes by year over the 6-year period, ensuring that the sum of the four themes for each academic year equals 100.

| Year | Themes | | | |
| --- | --- | --- | --- | --- |
|  | Foundation & liberal arts | Basic medical science | Clinical medicine | Humanities and social medicine |
| 1^st^ year |  |  |  |  |
| 2^nd^ year |  |  |  |  |
| 3^rd^ year |  |  |  |  |
| 4^th^ year |  |  |  |  |
| 5^th^ year |  |  |  |  |
| 6^th^ year |  |  |  |  |
